# Identification of macrophage related gene in colorectal cancer patients and their functional roles

**DOI:** 10.1186/s12920-021-01010-0

**Published:** 2021-06-13

**Authors:** Yingxiang Chen, Cui Zhang, Xiang Zou, Miao Yu, Bo Yang, Chen-Feng Ji, Shi-Yong Gao, Jun Li, Bin Liu

**Affiliations:** 1 Engineering Research Center for Medicine, Harbin University of Commerce, Harbin, 150076 China; 2 College of Pharmacy, Harbin University of Commerce, No. 138 Tongda Street, Harbin, 150076 Heilongjiang Province China

**Keywords:** Colorectal cancer, Macrophages, Macrophage-related genes

## Abstract

**Background:**

Recent scientific research has enabled the identification of macrophages related-genes (MaRG), which play a key role in the control of the immune microenvironment in many human cancers. However, the functional role of MaRGs in human tumors is ill-defined. Herein, we aimed at bioinformatically exploring the molecular signatures of MaRGs in colorectal cancer.

**Methods:**

A list of MaRGs was generated and their differential expression was analyzed across multiple datasets downloaded from the publicly available functional genomics database Gene Expression Omnibus. The weighted gene co-expression network analysis (WGCNA) was also applied to identify the partner genes of these MaRGs in colorectal cancer.

**Results:**

After integration of the results from analyses of different datasets, we found that 29 differentially expressed MaRGs (DE-MaRGs) could be considered as CRC-related genes as obtained from the WGCNA analysis. These genes were functionally involved in positive regulation of DNA biosynthetic process and glutathione metabolism. Protein–protein interaction network analysis indicated that PDIA6, PSMA1, PRC1, RRM2, HSP90AB1, CDK4, MCM7, RFC4, and CCT5 were the hub MaRGs. The LASSO approach was used for validating the 29 MaRGs in TCGA-COAD and TCGA-READ data and the results showed that ten among the 29 genes could be considered as MaRGs significantly involved in CRC. The maftools analysis showed that MaRGs were mutated at varying degrees. The nomogram analysis indicated the correlation of these MaRGs with diverse clinical features of CRC patients.

**Conclusions:**

Conclusively, the present disclosed a signature of MaRGs as potential key regulators involved in CRC pathogenesis and progression. These findings contribute not only to the understanding of the molecular mechanism of CRC pathogenesis but also to the development of adequate immunotherapies for CRC patients.

**Supplementary Information:**

The online version contains supplementary material available at 10.1186/s12920-021-01010-0.

## Background

Colorectal cancer is one of the deadliest tumors in the world [1]. The diagnosis of this tumor is often late due to the lack of appropriate screening methods. Also, the lack of global knowledge on the pathogenesis of colorectal cancer limits its effective management, which complicates the treatment options, leaving only surgical intervention, chemotherapy and radiotherapy as essential choices, all of which present a certain degree of side effects [2]. In addition, given the difference in the response of each patient to treatments and the differences in clinical presentation specific to each, a comprehensive study of the mechanisms involved in the pathogenesis and pathophysiology of colorectal cancer is strongly encouraged [3]. This is possible by elucidating the cellular and molecular machinery involved in the pathological process [4]. The immune response of patients plays a major role in the initiation and progression of human cancers [5]. Previous studies and numerous reviews have shown that weakened immune system is often accompanied by exacerbation of tumor progression [6–8]. In fact, immune cells have the ability to inhibit tumor growth and progression through a multitude of mechanisms combining the recognition and rejection of cancer cells. Other studies have shown that immune cells infiltrating tumors are of critical relevance in the tumor microenvironment (TME) [9–11]. Specifically, scientific research results suggest that cancer cells secrete inflammatory molecules such as cytokines and chemokines, the attraction of which promotes the infiltration of immune cells [12]. Based on this aspect, the researchers embarked on the development of drugs with immunotherapeutic properties. A recent example of such immunotherapies is the development of blockers of the interaction between PD-1 and PD-L1 [13]. However, it should be noted that cancer cells are capable of developing an ability to escape immune system by modulating the metabolism of immune cells such as T cells, macrophages and neutrophils [14]. The escape takes place through the regulation of a number of genes and proteins associated with these immune cells. Therefore, elucidating the molecular mechanisms involved in immune processes is fundamental not only for understanding cancer progression, but also for facilitating drug development and the implementation of appropriate therapeutic strategies.

Macrophages are immune cells that play an important role in antigenic degradation and the presentation of antigens [15]. Macrophages constitute an important class of immune cells in the cancerous microenvironment and their frequency is often associated with unfavorable patient survival [16]. Macrophages are involved in malignant processes such as cell invasion, angiogenesis and metastasis [17]. In colorectal cancer, macrophages play a primary role in liver metastasis [18]. Macrophages associated with tumors represent a regulatory bridge between cancer and the immune system of patients. Studies have shown that macrophages and genes that stimulate macrophages worsen the prognosis and condition of patients [19, 20]. Other studies suggest that macrophages are involved in the killing of immune cells and influence the effectiveness of different treatment strategies for cancers [21, 22]. Some studies, on the other hand, indicated that macrophages have the ability to kill cancer cells [23, 24]; therefore, it is evident that macrophages have both a pro- and anti-cancer properties. Previous studies have shown that CD68^+^-type macrophages associated with tumors have the potential to become a prognostic indicator for colorectal cancer [25, 26]. The multifunctional nature of macrophages in cancer pathogenesis and development could be attributed to the diverse regulatory roles of macrophage-related genes (MaRGs). Representative MaRGs include IL-6 (Interleukin 6), IL-8 (Interleukin 8), CD80 (Cluster of differentiation 80), and PIM1 (Pim-1 Proto-Oncogene, Serine/Threonine Kinase) [27]. IL-6 was previously proposed as a preoperative serum marker for predicting colon cancer prognosis [28]; it is also involved in promoting the stemness of colon cancer by provoking inflammation [29]. IL-8 was found significantly associated with the tumor size, stage and liver metastasis of colon cancer [30], which might be ascribed to its antigenic property that promotes colon cancer metastasis and supports tumor growth [31]. CD80 is recognized as an important co-stimulatory molecule responsible for eradication of tumor cells; in colon cancer, CD80 coordinates the immune surveillance for precancerous lesions [32]. PMI1 is a well-established oncogene whose overexpression in colon cancer could counteract the deprivation of glucose by triggering a compensatory Warburg effect, conferring colon cancer with survival advantages under metabolic stress [33]. The above findings suggest a sophisticated interplay between MaRGs and colon cancer that determine the progression and prognostic outcomes of colon cancer; however, there are still considerable MaRGs and the corresponding mechanisms still remain to be uncovered in the context of colon cancer. In addition, research on the interactions between macrophages and cancer cells that would allow the discovery of new genetic signatures of macrophages as prognostic and therapeutic markers is lacking with regard to CRC. In this regard, further investigation on multifarious facets of MaRGs is required.

Bioinformatics is a discipline involving the use of computational tools for the *in-silico* analysis of biological data. The advances in genomics and transcriptomics have accelerated the development of this field which has allowed the extraction of valuable biological information from experimentally-derived data or publicly available data. This technique has been applied for retrieving significant data for various diseases including diabetes, neurodegenerative diseases, cardiovascular diseases and cancers [34]. However, bioinformatical analysis of the implications of MaRGs in colorectal cancer has not been reported so far.

Thus, in the present study, we set out to analyze the importance of MaRGs in colorectal cancer using bioinformatics tools based on publicly available data.

## Methods

### Datasets collection

GeneCard (https://www.genecards.org/) is a comprehensive human gene database that contains all annotated and predicted human genetic information. We downloaded 119,923 macrophage-related genes (MaRGs) based on the keyword "macrophage" from GeneCard. The corresponding information of MaRGs is available in Additional file 6: Table S1. We screened all microarray datasets related to CRC in the Gene Expression Omnibus database (GEO, https://www.ncbi.nlm.nih.gov/geo/). The keywords “colorectal cancer” and “gene expression profiling” were used to query the datasets from the GEO database. The datasets conform to the following criteria were reserved: (I) the organism was “Homo sapiens”; (II) the experiment types were “Expression profiling by array”; (III) the dataset contained both tumor and control samples; (IV) the annotation of the gene probes were completed; (V) the number of normal or control samples was larger than 5. Finally, we selected five microarray datasets including 135 normal samples and 167 tumor samples, and the detailed information can be accessed in Table 1.

**Table 1:** Information of five datasets form GEO database

Country	Organization	Series	Platform	Normal	Tumor
Italy	University of Torino	GSE23194	GPL570	12	5
Japan	Juntendo University	GSE32323	GPL570	17	17
USA	Roche Innovation Center New York	GSE103512	GPL13158	12	57
China	Sun Yat-sen University	GSE156355	GPL21185	6	6
Italy	Fondazione IRCCS Istituto Nazionale dei Tumori	GSE37182	GPL6947	88	82

In addition, according to the keywords "TCGA-COAD" and "TCGA-READ", we downloaded two datasets from The Cancer Genome Atlas Program (TCGA, https://www.cancer.gov/about-nci/organization/ccg/research/structural-genomics/tcga). The TCGA-COAD dataset contains 20 normal samples and 436 tumor samples and TCGA-READ dataset contains 6 normal samples and 161 tumor samples, which were used to validate the efficacy of genes.

### Identification of differentially expressed MaRGs (DE-MaRGs)

When a gene symbol was mapped to multiple probes, the average expression of this gene was preserved whereas genes containing missing values (or zero values) were removed. The R “limma” package [35] was used for data preprocessing and differential expression analysis. Quantile method for data normalization and logarithmic transformation for data scaling was performed in R. We screened out differentially expressed genes (DEGs) from each dataset with a threshold of |logFC|> 0.263 and P-value < 0.05, and obtained common DEGs that were up-regulated and down-regulated in the 5 datasets through merging. Then weighted gene co-expression network analysis (WGCNA) was performed to analyze the relationship between gene co-expression modules and clinical traits in each dataset. The correlation between CRC and modules was calculated by the Pearson Correlation Coefficient (PCC) between eigengenes per module and CRC status. The two modules with the highest correlation (positive correlation and negative correlation) with CRC status of each dataset were retained. CRC-related genes (CrRGs) were obtained by the intersection of the genes of modules most significantly associated with CRC after WGCNA analysis of the five datasets. Finally, we combined the MaRGs, DEGs, and CrRGs, and obtained DE-MaRGs that may be potential biomarkers in CRC. The R “WGCNA” package [36] was used to perform WGCNA in this study.

### Functional enrichment analysis of DE-MaRGs

To reveal the biological functions of the DE-MaRGs, we performed the Gene Ontology (GO) and Kyoto Encyclopedia of Genes and Genomes (KEGG) pathway enrichment analysis via the R “clusterProfiler” package [37]. The terms with adjusted P-value 0.05 were considered significant and the top 20 terms were visualized via R “ggplot2” package [38].

### Protein–protein interaction (PPI) network construction and identification of hub DE-MaRGs

The Search Tool for The Retrieval of Interacting Genes/Proteins (STRING, https://string-db.org/cgi/) is a database of known and predicted protein–protein interactions. Herein, we uploaded DE-MaRGs to the STRING database and selected the organism "Homo sapiens". TSV file was downloaded from the STRING database and used as network input for visualization of the PPI network in the Cytoscape software 3.7.2. We identified the hub DE-MaRGs in the PPI network via the Cytoscape plugin MCODE with parameters as degree cut-off ≥ 2, node score cut-off ≥ 0.2, k-core ≥ 2, and max depth = 100. Then, we analyzed the types of mutations and the mutation rate that may exist in the key DE-MaRGs using the R Maftools package based on TCGA- COAD and TCGA- READ mutation data information. In this study, COAD and READ mutation information was downloaded from the TCGA database with the key word “TCGA-COAD” or “TCGA- READ”, “Somatic Variant Aggregation and Masking”, and “Simple nucleotide variation”.

### Identification of the key DE-MaRGs

Based on the TCGA-COAD and TCGA-READ datasets, we used the least absolute shrinkage and selection operator (LASSO) to obtain the key DE-MaRGs. The R “glmnet” package [39] was used to implement LASSO analysis. Since the normal samples were too small compared to tumor samples, it may not be statistically valid to use two full TCGA datasets for validation. Herein, we performed LASSO analysis repeatedly on a random subset of tumor samples and all of the normal samples (with tumor-normal ratio of 3:1) and then the overlapped results were used as valid results. The time of LASSO analyses was set as 1000. DE-MaRGs were counted every time they were identified by LASSO analysis, and the eight DE-MaRGs with the highest cumulative number were selected as candidate genes. The key DE-MaRGs were obtained by the intersection of the candidate genes identified from the TCGA-COAD and TCGA-READ dataset.

To investigate the prognosis effects of the key DE-MaRGs, we performed survival analysis. The normal samples in the TCGA-COAD and TCGA-READ datasets were excluded, and the relationship between the key prognosis DE-MaRGs and the survival time of CRC patients was analyzed using the R "survival" package (https://cran.r-project.org/web/packages/survival/index.html). Kaplan–Meier Curve analysis was used to obtain overall survival (OS) and disease-free survival (RFS) of CRC patients. Based on the different tumor stages of CRC, we obtained the relationship between the expression of the key DE-MaRGs and the tumor stage. In order to verify the mutation information of key DE-MaRGs, we constructed a CRC mutation prediction model. The mutation data were downloaded as described above and using the R "Maftools" package (https://cran.r-project.org/web/packages/maptools/index.html), we analyzed the types of mutations and the mutation rate of the key DE-MaRGs based on the mutation data.

### Evaluation of clinical independence and construction of the nomogram

We deleted CRC samples with missing clinical information, including survival status, time, tumor stage, age, sex, and weight, from the TCGA-COAD and TCGA-READ datasets. R "rms" package was used to construct univariate and multivariate Logistic Regression analyses for CRC clinical information. Then we constructed the Cox risk model using the R "survival" package and R "rms" package (https://cran.r-project.org/web/packages/rms/index.html). Finally, we used the R "rms" package to integrate clinical information for nomogram construction. Ten key DE-MaRGs model was constructed based on TCGA-COAD and TCGA-READ datasets according to gene expression profiles. Moreover, ROC curves were used to estimate the prediction capabilities of the key DE-MaRGs and were implemented by R “ROCR” package.

## Results

### Identification and functional enrichment of the DE-MaRGs in five CRC datasets

**Fig. 1:**
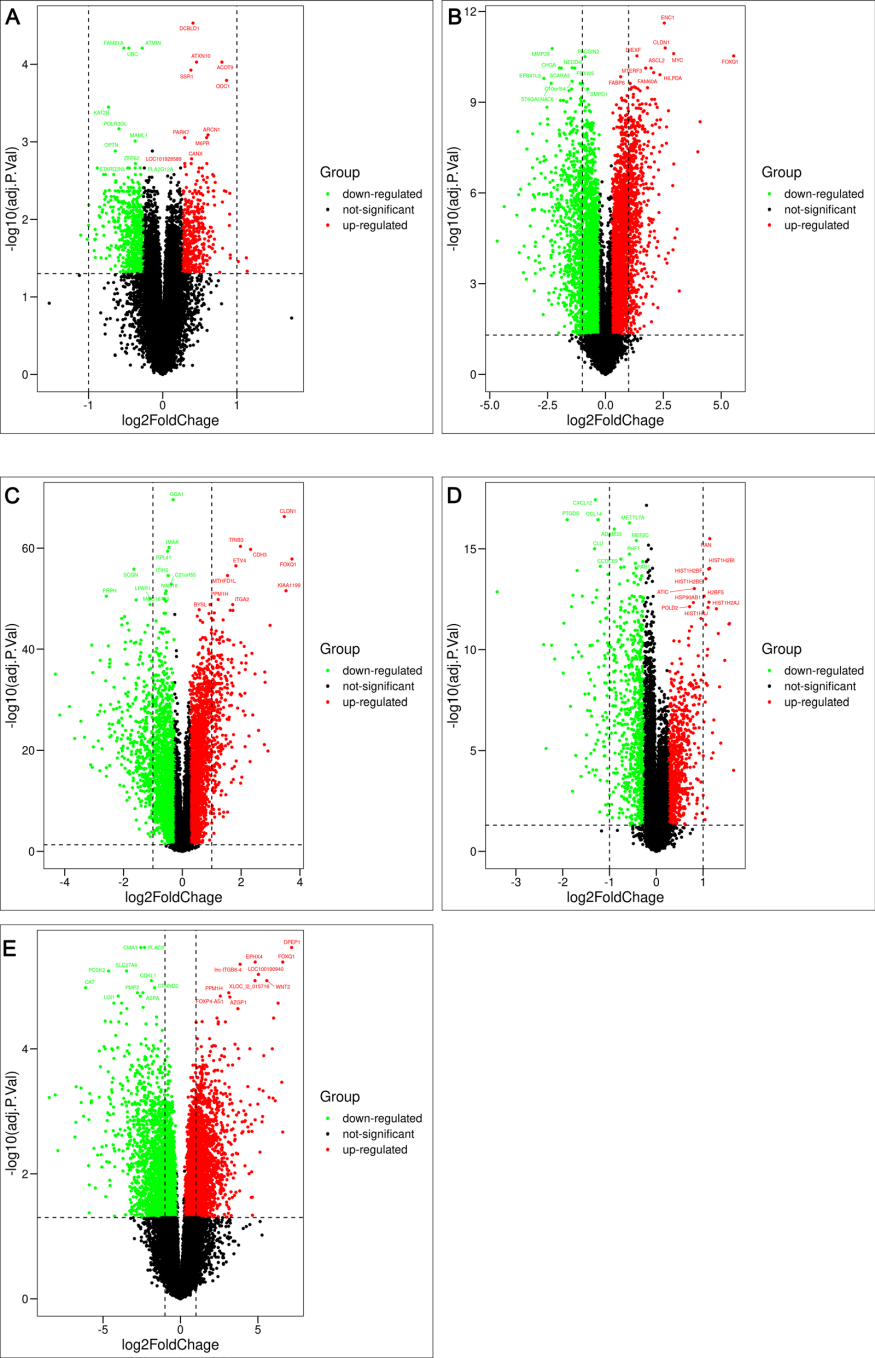
The volcano plots of DEGs between CRC and normal group. **a** Volcano plot of DEGs identified in GSE23194. **b** Volcano plot of DEGs identified in GSE32323. **c** Volcano plot of DEGs identified in GSE37182. **d** Volcano plot of DEGs identified in GSE103512. **e** Volcano plot of DEGs identified in GSE156355

**Fig. 2:**
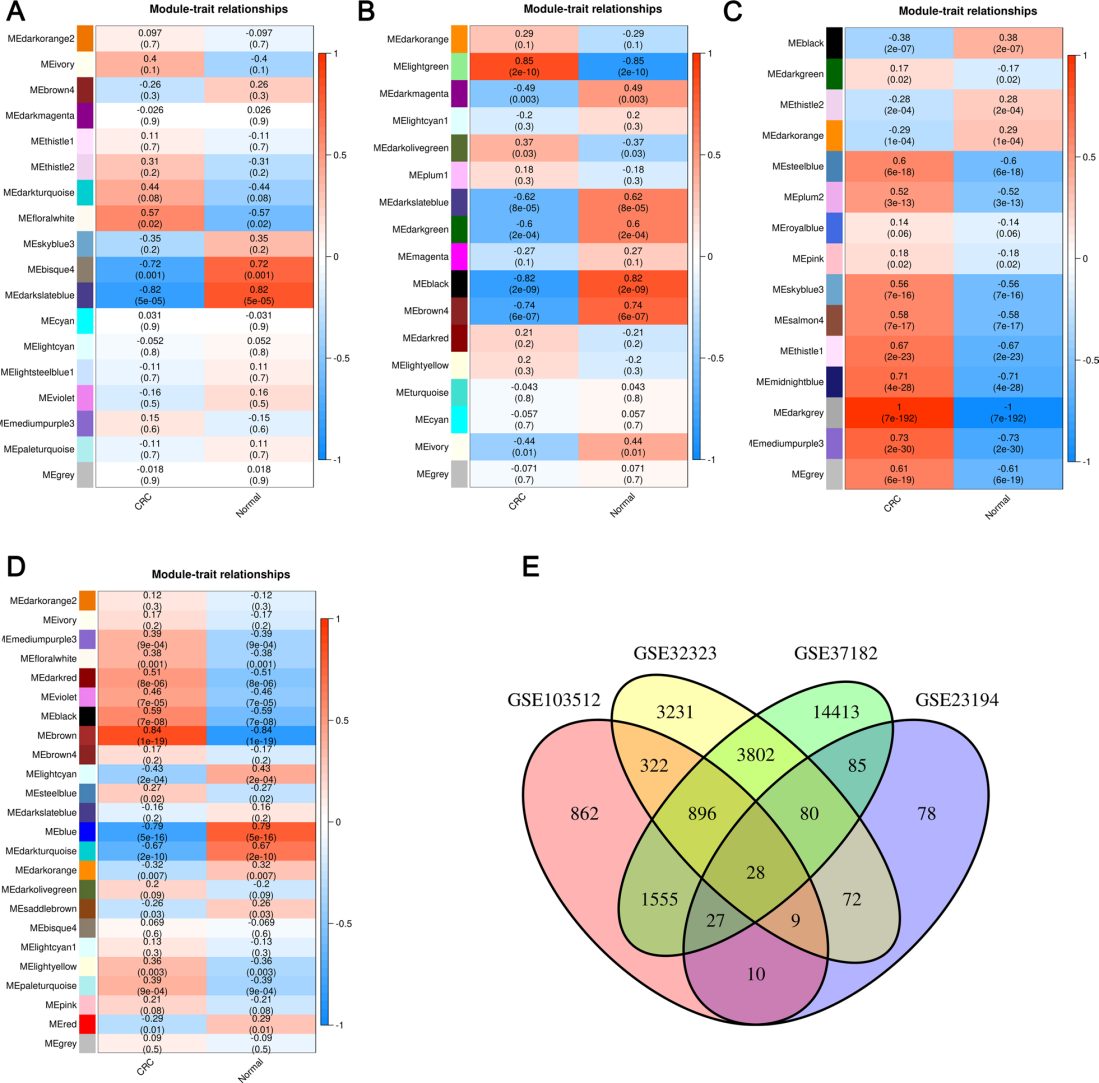
Identification of CrRGs. **a** Module-trait relationships between CRC and modules in GSE23194. **b** Module-trait relationships between CRC and modules in GSE32323. **c** Module-trait relationships between CRC and modules in GSE37182. **d** Module-trait relationships between CRC and modules in GSE103512. **e** Venn plot of genes in modules associated with CRC in four datasets

**Fig. 3:**
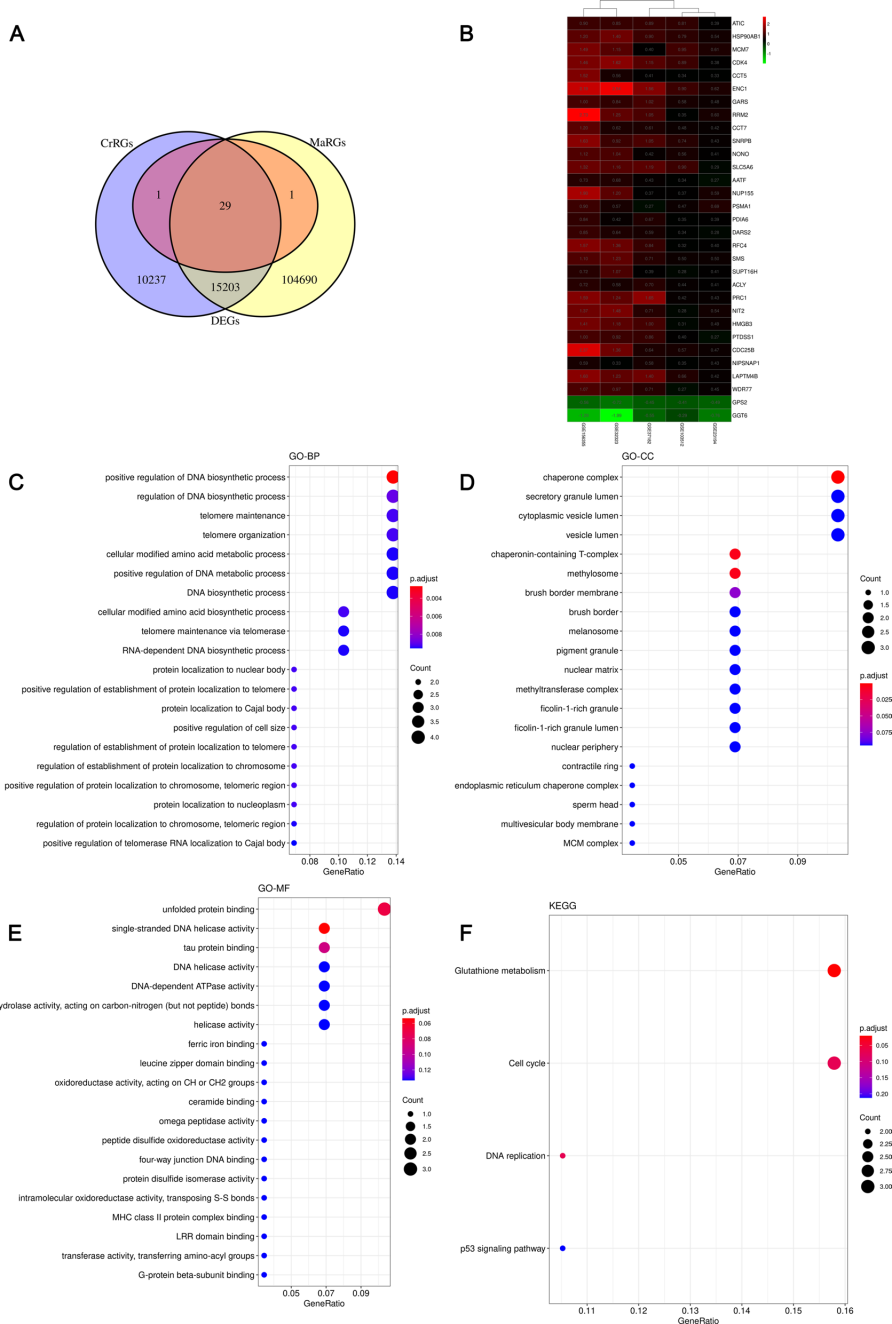
Identification and function enrichment of DE-MaRGs. **a** Venn plot of MaRGs, DEGs, and CrRGs. **b** The heatmap of DE-MaRGs in five datasets with log2FC in the colored box. **c–f** GO analysis and KEGG pathway analysis of DEG-MaRGs. The size of the circle represents the number of enriched genes, and the color is related to the *P*-value

To identify the DE-MaRGs, we performed differential expression analysis based on five CRC-related datasets from the GEO database. The volcano plot for DEGs in the five datasets and heatmap of the top 10 up-regulated DEGs and down-regulated DEGs in the five datasets were shown in Fig. 1 and Additional file 1: Figure S1, respectively. After integrating the five CRC datasets, we obtained a total of 29 up-regulated CRC DEGs and two down-regulated CRC DEGs. Since the GSE156355 dataset did not conform to a scale-free distribution, we only analyzed the remaining four datasets separately with the WGCNA approach (Additional files 2, 3, 4, 5: Figs. S2–S5). The module-traits relationship heatmap of the four datasets was shown in Fig. 2a–d. A total of 8 modules related to CRC were screened, and 25,470 of CRC-related genes (CrRGs) were obtained after merging these modules (Fig. 2e). According to the keyword "macrophage", we downloaded a list of 119,923 MaRGs in GeneCard. After the intersection of MaRGs, DEGs, and CrRGs, 29 DE-MaRGs were obtained (Fig. 3a), and the heatmap of the DE-MaRGs in the five datasets based on log2FC was shown in Fig. 3b.

GO and KEGG analyses were used to reveal the biological functions of the 29 DE-MaRGs. We found that the 29 DE-MaRGs were mainly enriched in positive regulation of DNA biosynthetic process, telomere maintenance, cellular modified amino acid metabolic process, chaperone complex, chaperon-containing T-complex, methylome. Moreover, glutathione metabolism and DNA replication were considered as the significant pathway of 29 DE-MaRGs via KEGG pathway analysis. The bubble charts of GO and KEGG are shown in Fig. 3c–f.

### The PPI network of 29 DE-MaRGs and hub gene mutation analysis

**Fig. 4:**
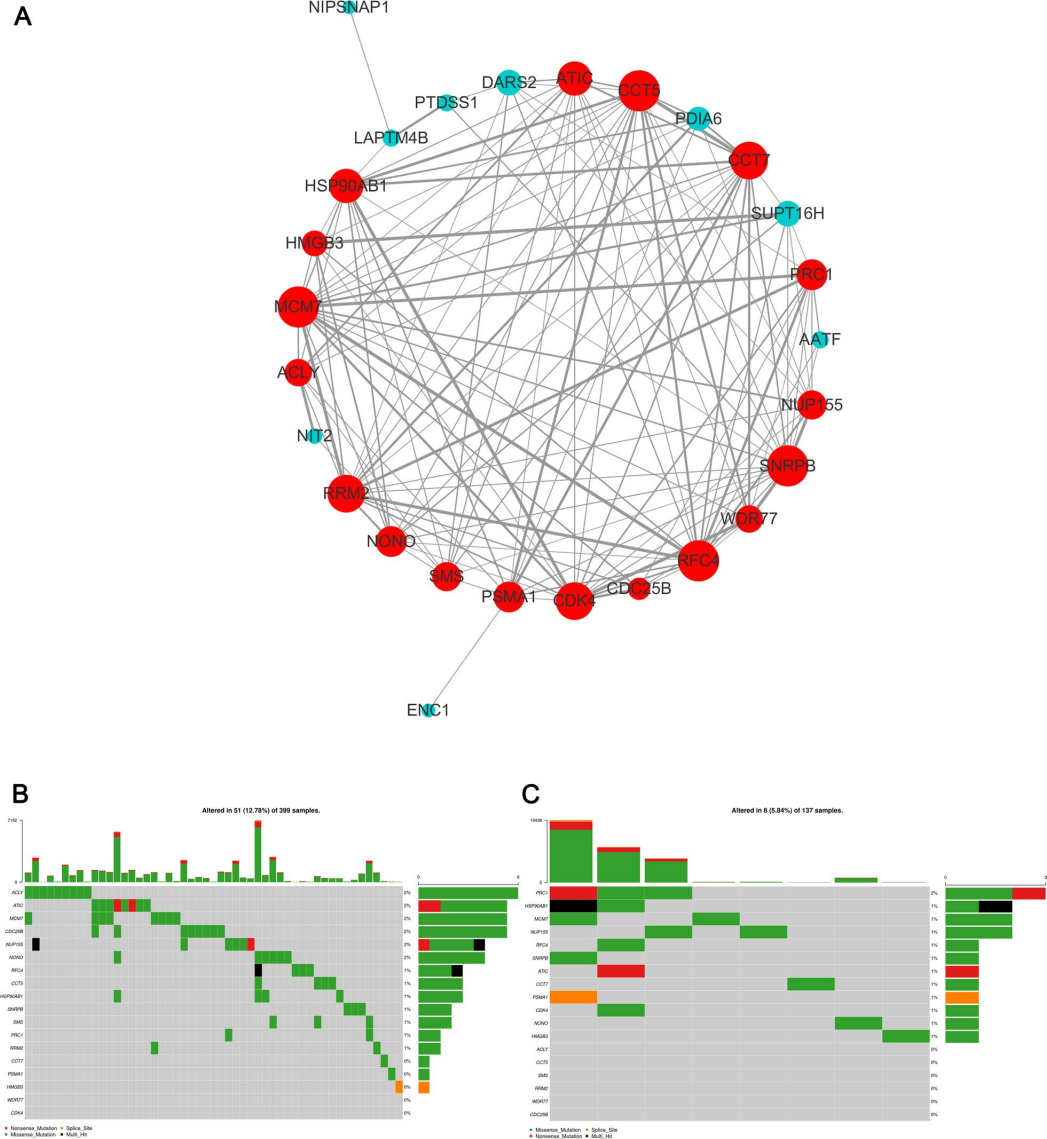
Protein–protein network of the hub DE-MaRGs and maftools analysis. **a** The interaction network of the DE-MaRGs. **b** The gene mutation overview of 18 hub genes in TCGA CRC patients based on TCGA-COAD mutation data. **c** The gene mutation overview of 18 hub genes in TCGA CRC patients based on TCGA-READ mutation data

Through the STRING website, we constructed a PPI network composed of 29 DE-MaRGs-encoded proteins. After removing the proteins that were not connected to the network, the final PPI network contained 27 nodes and 130 edges (Fig. 4a). The top 18 hub proteins with the highest degrees were RFC4, PRC1, SNRPB, ACLY, ATIC, CCT7, CCT5, SMS, HSP90AB1, PSMA1, RRM2, MCM7, WDR77, CDK4, NUP155, NONO, HMGB3, and CDC25B. More detailed information can be found in the Additional file 7: Table S2. The mutation analysis results (Fig. 4b) showed that ACLY, ATIC, MCM7, and CDC25B were the genes with the highest mutation frequency in TCGA-COAD mutation data. Moreover, NUP155 RFC4 presented multiple mutations in one sample. Similarly, we also found that the mutation frequency of PRC1 was highest in the TCGA-READ mutation data. However, there was no tendency for ACLY, CCT5, SMS, RRM2, WDR77, and CDC25B to mutate in the TCGA-READ mutation data (Fig. 4c).

### Identification of the key DE-MaRGs

LASSO analysis was used to screen out the key DE-MaRGs from the 29 DE-MaRGs using R "glmnet" package (https://cran.r-project.org/web/packages/glmnet/index.html). With a 1000-times resampling and training set: testing set ratio of 3:1, the LASSO validation selected 8 candidate genes (SUPT16H, ENC1, PSMA1, HSP90AB1, PRC1, WDR77, AATF, and NUP155) from the TCGA-COAD dataset, and another 8 candidate genes (SUPT16H, ENC1, PSMA1, ATIC, PRC1, WDR77, NUP155, and NIT2) from TCGA-READ dataset. After deduplication of the two sets of candidate genes, ten key DE-MaRGs (SUPT16H, ENC1, PSMA1, ATIC, PRC1, WDR77, NUP155, NIT2, HSP90AB1, and AATF) were finally obtained. The information of the total count of each key DE-MaRG (number of times it had a non-zero coefficient) in 1000-times LASSO selection was provided in Additional file 8: Table S3.

**Fig. 5:**
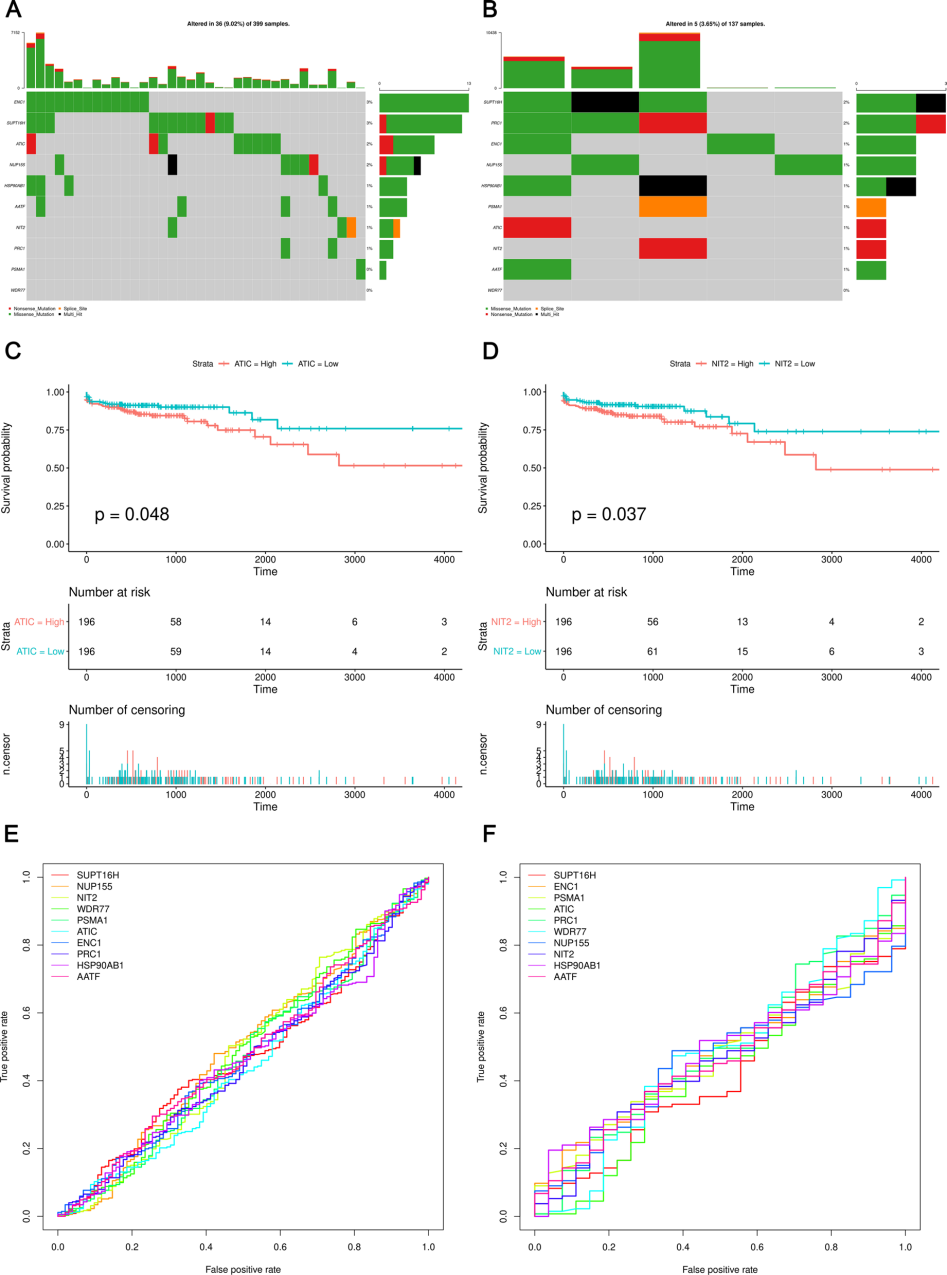
Validation of the efficacy for the ten key DE-MaRGs. **a** The gene mutation overview of key DE-MaRGs based on TCGA-COAD mutation data. **b** The gene mutation overview of key DE-MaRGs based on TCGA-READ mutation data. **c** KM curve analysis of ATIC (*P*-value < 0.05) based on the TCGA-COAD dataset. **d** KM curve analysis of NIT2 (*P*-value < 0.05) based on the TCGA-COAD dataset. **e** ROC curves of key DE-MaRGs model based on the TCGA-COAD dataset. **f** ROC curves of key DE-MaRGs model based on the TCGA-READ dataset

The mutation information of ten key DE-MaRGs was predicted by the CRC simple nucleotide variation data downloaded from the TCGA database. Figure 5a showed that ENC1, SUPT16H, and ATIC were the top mutant genes in the TCGA-COAD mutation dataset. The mutation types of these genes were mainly missense mutations. In the TCGA-READ mutation dataset, the mutation types of DE-MaRGs were more diverse. Figure 5b showed that the mutation type of PSMA1 was splice site, and SUPT16H, PRC1, and NUP155 were the top-ranked mutant genes. In addition, SUPT16H was considered to have multiple mutations in the same sample, and its mutation frequency reached 2%. Except for NIT2 (P-value < 0.05) and ATIC (P-value < 0.05) (Fig. 5c, d), no difference was found between the results from the survival status, survival time, and KM curve analysis of the high expression status subgroup and low expression status group in key DEG-MaRGs. ROC curve analysis (Fig. 5e, f) revealed that the area under the ROC curve (AUC) of the ten key DE-MaRGs models were close to 0.5.

### Nomogram building and validation

**Fig. 6:**
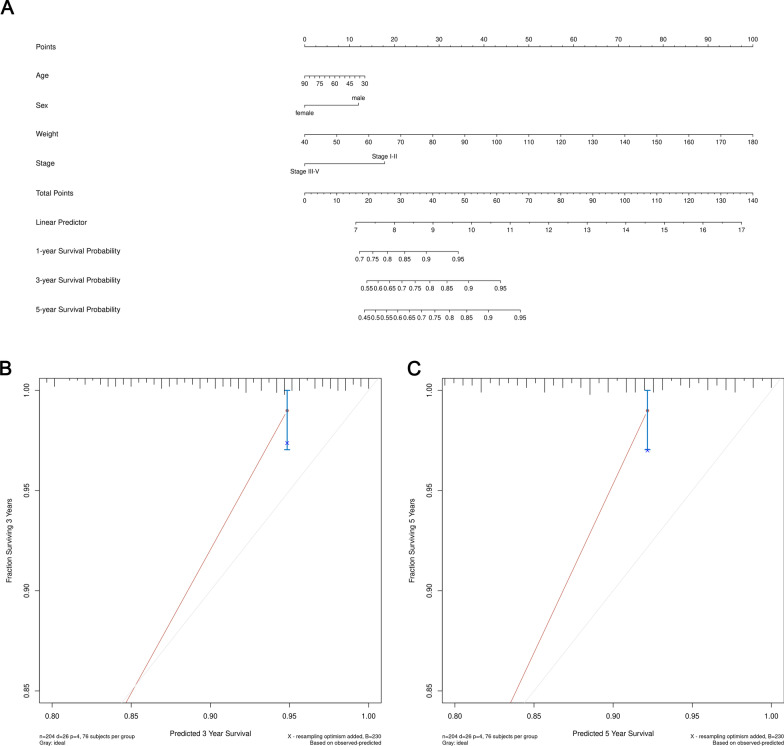
Nomogram for predicting the 3-year and 5-year survival probability of patients with CRC based on TCGA-COAD dataset. **a** Prognostic nomogram for CRC patients. **b** Calibration curve for the nomogram at 3-year. **c** Calibration curve for the nomogram at 5-year

**Fig. 7:**
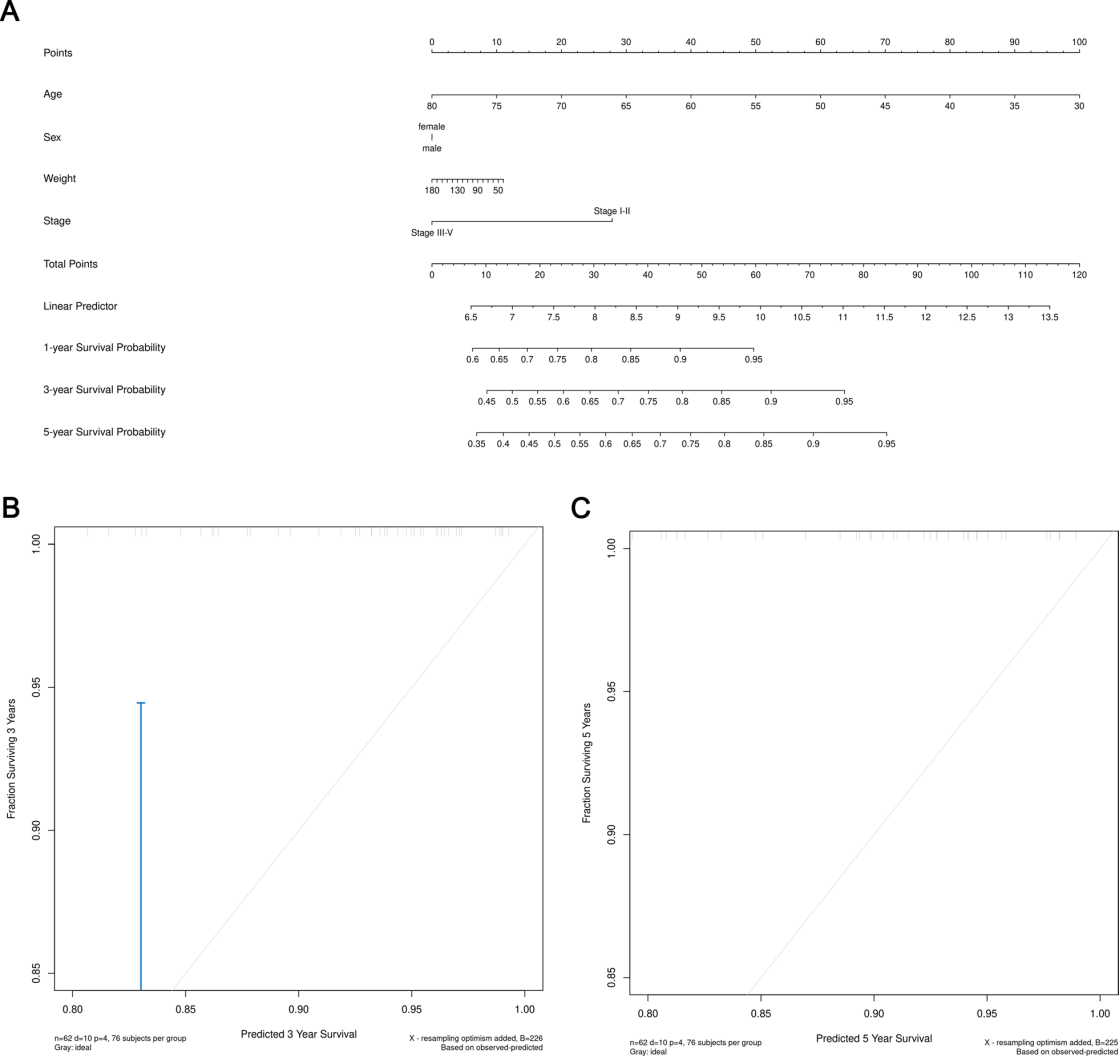
Nomogram for predicting the 3-year and 5-year survival probability of patients with CRC based on TCGA-READ dataset. **a** Prognostic nomogram for CRC patients. **b** Calibration curve for the nomogram at 3-year. **c** Calibration curve for the nomogram at 5-year

According to the patient's clinical information, we constructed a comprehensive prognostic array map based on the entire TCGA data set to assess the probability of survival of CRC patients within 3 and 5 years. Six clinical features, including survival status, time, tumor stage, age, sex, and weight, were included in the nomogram analysis (Fig. 6a, 7a). In the TCGA cohort, in terms of 3-year and 5-year survival rates, the calibration chart showed good agreement between nomogram predictions and actual observations (Fig. 6b, c, 7b, c).

## Discussion

Macrophage is a type of innate immune cell that plays an important role in host defense and inflammation. They are highly plastic and can be polarized into subtypes with different functions in different pathological environments. Tumor-associated macrophages (TAMs) are abundant in the TME, and they play an important role in promoting the growth of various tumors [40]. It is worth noting that different types of TAM have different effects on the TME of CRC. For example, some macrophages can promote tumor formation, while others inhibit tumor formation. High macrophage infiltration is believed to be related to the prognosis of tumors [41]. Although macrophage is one of the most common cells in the microenvironment of colorectal cancer, their prognostic role in tumors is not fully understood [42]. The differentiation and activation of macrophages require regulation of gene expression, which is subject to the interaction of many factors, including transcription factors and epigenetic modifications [43]. For example, there are differences of the human c-fes gene and murinespi-1 (PU.1) gene in constitutive and inducible gene expression in macrophages [44]. In solid tumors, many macrophages and other immune cells constitute the TME [45]. The TME changes the malignancy of tumors. Studies have shown that macrophages stimulate tumor cell migration, invasion, vascular invasion and strengthen blood vessels to promote the development of tumors in the direction of malignant tumors [46, 47]. Generally, the expression level of genes is specific in TAMs and tumor cells. However, the expression of some genes is consistent in tumor cells and TAMs. Myeloma cells can secrete vascular endothelial growth factor A (VEGFA) to stimulate angiogenesis [48]. A recent study reported that M2 macrophages and RPMI 8226 cells can synergistically promote the proliferation, migration and tube formation of human umbilical vein endothelial cells (HUVEC), and the consumption of VEGFA in both cell types can inhibit the tube formation ability of HUVEC [49]. In addition, representative molecular markers for macrophage such IL-6, IL-8, CD80, and PIM1 were closely associated with CRC [28–33]. Therefore, we speculate that there are other unidentified macrophage-related genes that bear the potential to regulate the occurrence and metastasis of CRC. In this study, we integrated five CRC transcriptome datasets, including 302 samples, and screened the mMaRGs in CRC. Through differential expression analysis, we obtained 31 DEGs from the five datasets, 29 of which were up-regulated in the five datasets while two of them were down-regulated. We identified 25,470 CrRGs from the four datasets (GSE23194, GSE32323, GSE37182, and GSE103512) via WGCNA analysis. Then 119,923 MaRGs were obtained from GeneCard with the search keyword “macrophage”. Finally, we obtained 29 DE-MaRGs via merging DEGs, CrRGs and MaRGs.

At present, the role of macrophages in the pathogenesis of CRC is still unclear. At the molecular level, the activation of oncogenes and the inactivation of tumor suppressor genes are related to the occurrence of CRC [50]. At the cellular level, macrophages in the TME may adopt different polarization states, thereby affecting the occurrence of CRC. The regulation and expression of genes promote the differentiation and activation of macrophages, which is affected by the interaction of many factors, including transcription factors and epigenetic modifications [43]. Therefore, it is very meaningful to study the potential biological functions and pathways of the genes associated with macrophages in CRC. We found that the main biological pathways of the 29 DE-MaRGs enrichment were DNA biosynthetic process, telomere maintenance, cellular modified amino acid metabolic process, chaperone complex. Telomere maintenance is an important sign of cancer. Continuous classification of cells can cause telomere shortening, but tumor cells can use telomere maintenance mechanisms to avoid this phenomenon. The presence of TAMs will shorten the survival time of patients. A previous study pointed out that most tumors with uncertain mechanisms of telomere maintenance have a large number of TAMs [51], which is consistent with our finding that these DE-MaRGs were enriched in the telomere maintenance. Activated macrophages can be divided into M1 macrophages and M2 macrophages. Our finding demonstrated that the cellular modified amino acid metabolic process was a significant pathway for DE-MaRGs, which can explain the M2 polarization defect and the enhancement of M1 polarization caused by Lamtor1 deficiency, amino acid starvation, and mTOR inhibition [52]. Intracellular macrophage migration inhibitory factor (MIF) usually becomes stable in human cancer cells. MIF can promote tumor cell survival. We found that DE-MaRGs were enriched in the chaperone complex, which is consistent with a previous study reporting that tumor-activated HSP90 chaperone complex can protect MIF from degradation [53]. It is worth noting that molecular chaperone proteins are pleiotropic signals of many kinds of cells. Henderson and colleagues [54] put forward a hypothesis by comparing the literature, that is, animal molecular chaperones can induce a variety of macrophage activation states. We found that glutathione metabolism was an important pathway for DE-MaRGs. The oxidative state of cells is one of the key factors that mediate apoptosis, and glutathione plays a vital role in mediating cell apoptosis through NO* and reactive oxygen species (ROS). Thus, our findings can explain why glutathione levels determine apoptosis in macrophages [55]. In another study, glutathione was considered as a significant protective component against NO cytotoxicity on macrophages [56]. In conclusion, our findings can provide new ideas on the functions and pathways of the genes related to the macrophages and CRC.

To better understand the pathogenesis mechanisms of CRC and the related function of macrophages, we studied the interaction of proteins encoded by DE-MaRGs. Here, we used PPI analysis to construct a network of 22 nodes and 48 edges. After Cytoscape MCODE analysis, we finally obtained nine hub DE-MaRGs, including PDIA6, PSMA1, PRC1, RRM2, HSP90AB1, CDK4, MCM7, RFC4, and CCT5. The mutation results showed that MCM7, HSP90AB1, and RFC4 were the genes with the highest mutation frequency. A previous study demonstrated that RFC4 is overexpressed in CRC and is associated with tumor progression and poor survival results [57], which is consistent with the results found in this study that RFC4 was jointly up-regulated in the five datasets. RFC is involved in DNA replication as a clamp loader and is regulated in a series of cancers. According to the results of Cytoscape MCODE analysis, we found that PDIA6 may play an important role in macrophages. A study reported that oxysterol loaded in THP-1 macrophages caused a decrease in the abundance of proteins related to cell death or cell life, including PDIA6 [58], which is consistent with the findings of the present study. At present, there is no sufficient evidence that PRC1 is directly related to macrophages, but a previous study reported that PRC1 had a regulatory role in immune evasion and angiogenesis [59]. Recent studies have shown that overexpression of PRC1 may promote the formation of various tumors, including ovarian cancer [60] and colorectal cancer [61]. The expression of RRM2 and p53R2 is related to the malignancy and progression of several types of tumors. Overexpression of RRM2 was thought to be useful for predicting metastasis and disease prognosis [62]. Similarly, the ribonucleotide reductase subunit RRM2B was considered to be associated with advanced stage III-IV tumors that have better survival than early stage I-II tumors, and its expression was associated with better survival prognosis in CRC patients [63]. However, the mechanism of how macrophages regulate the occurrence and metastasis of CRC through RRM2B is still unclear. We suggested that CDK4 may be indirectly involved in the pathway through which macrophages promote the formation of CRC. CDK4 is a type of cyclin-dependent kinase, its inhibitor gene p16 (INK46a) can inhibit rheumatoid arthritis in synovial tissue, in which macrophages are the main source of inflammatory cytokines [64]. CDK4 is the basic driving factor of the cell cycle and is essential in the initiation and development of various malignant tumors. A previous study reported that selective CDK4 inhibitors can induce tumor cell cycle arrest and promote anti-tumor immunity. Therefore, macrophages may participate in the pathogenesis and development of CRC by indirectly regulating the expression of CDK4 [65]. Although we have discussed some genes related to macrophages and CRC, there are still many genes that have not yet been reported. In the future, more in vitro and in vivo experiments are needed to verify the role of these genes in macrophages in the occurrence of CRC.

In order to further study the prognostic role of the 29 DE-MaRGs in CRC, we used LASSO analysis to select 10 key DE-MaRGs from the 29 DE-MaRGs. Among the key DE-MaRGs, we found that the expression levels of NIT2 (P-value < 0.05) and ATIC (P-value < 0.05) were related to the prognosis of CRC via survival analysis. A previous study indicated that the down-regulation of NIT2 inhibited the proliferation of colon cancer cells through the caspase-3 and PARP pathways, and induced cell cycle arrest [66]. Another research team gave a similar finding that NIT1 inhibited the growth of CRC through the positive feedback formed by NIT1 and the activation of the TGFβ-Smad signaling pathway [67]. Therefore, we anticipate that a low level of NIT2 may be associated with a better CRC prognosis. In addition, our study found that the expression level of NIT2 was up-regulated in the five CRC datasets, indicating that the high expression level of NIT2 may be related to the occurrence of CRC. As far as we know, we found for the first time that low level of ATIC was associated with better CRC prognosis, which is consistent with the increase of ATIC in the five CRC datasets. The mechanism of how ATIC is involved in CRC and the correlation between ATIC and macrophages deserves more in-depth research.

Although we have discovered some key DE-MaRGs and discussed how these genes participate in the formation and development of CRC, there are still some genes that have not been reported, and more in vivo and in vitro experiments are needed to verify their functions. We have discovered several possible pathways in which DE-MaRGs participate; this can provide new ideas for understanding the pathways via which macrophages may participate in CRC. However, the present study was only based on the computational analysis of biological information, and more studies are needed to verify our findings in the future. Moreover, we evaluated the predictive effects of ten key DE-MaRGs models and constructed the nomogram of CRC using the TCGA dataset. Due to the limited number of samples of the dataset we used, these CRC prognostic models may have more room for improvement in the future. There are few published macrophage microarray data, thus we used CRC datasets to explore the role of MaRGs in CRC pathogenesis, which can explain the specific role of these genes in CRC and indirectly give insights on the role of macrophages in CRC. In the future, we will collect macrophages associated with CRC for single-cell sequencing to get insight into the specific molecular mechanism of macrophages in the occurrence and development of CRC.

## Conclusion

We obtained 29 DE-MaRGs associated with macrophages and CRC from five CRC datasets through a comprehensive analysis method. These genes may directly or indirectly participate in the occurrence and development of CRC through telomere maintenance, cellular modified amino acid metabolic process, chaperone complex, etc. Among these genes, NIT2 and ATIC were considered to be related to the prognosis of CRC (P-value < 0.05). In the future, we will conduct in vivo and in vitro experiments to verify the role of these genes in macrophages and CRC. Our research provides a new direction for understanding the biological functions of the genes related to both macrophages and CRC, and provide more diverse options for the prognosis of CRC.

## Supplementary Information

**Additional file 1:** **Fig. S1.** Heatmap of the top ten DEGs of up-regulated cluster and down-regulated cluster in five datasets. (A) Heatmap of DEGs identified in GSE23194. (B) Heatmap of DEGs identified in GSE32323. (C) Heatmap of DEGs identified in GSE37182. (D) Heatmap of DEGs identified in GSE103512. (E) Heatmap of DEGs identified in GSE156355

**Additional file 2:** **Fig. S2.** WGCNA analysis of GSE23194. (A) Sample dendrogram and clinical trait heatmap. (B) The identification of βvalue for the optimal scale-free topology network. (C) Module identification. The dendrogram indicates the gene clustering according to TOM dissimilarity

**Additional file 3:** **Fig. S3.** WGCNA analysis of GSE32323. (A) Sample dendrogram and clinical trait heatmap. (B) The identification of βvalue for the optimal scale-free topology network. (C) Module identification. The dendrogram indicates the gene clustering according to TOM dissimilarity

**Additional file 4:** **Fig. S4**. WGCNA analysis of GSE37182. (A) Sample dendrogram and clinical trait heatmap. (B) The identification of βvalue for the optimal scale-free topology network. (C) Module identification. The dendrogram indicates the gene clustering according to TOM dissimilarity

**Additional file 5:** **Fig. S5.** WGCNA analysis of GSE103512. (A) Sample dendrogram and clinical trait heatmap. (B) The identification of βvalue for the optimal scale-free topology network. (C) Module identification. The dendrogram indicates the gene clustering according to TOM dissimilarity

**Additional file 6:** **Table S1.** The detailed information of MaRGs obtained from GeneCard with the keyword "macrophage"

**Additional file 7:** **Table S2.** The detailed information about the PPI analysis of 29 DE-MaRGs

**Additional file 8: Table S3. **The detailed information of the LASSO analysis for identification of key DE-MaRGs

## Data Availability

The datasets GSE156355, GSE23194, GSE32323, GSE37182 and GSE103512 analyzed during the current study are available in the Gene Expression Omnibus repository. Persistent web links to these datasets are as follow: GSE156355: https://www.ncbi.nlm.nih.gov/geo/query/acc.cgi?acc=GSE156355; GSE23194: https://www.ncbi.nlm.nih.gov/geo/query/acc.cgi?acc=GSE23194; GSE32323: https://www.ncbi.nlm.nih.gov/geo/query/acc.cgi?acc=GSE32323; GSE37182: https://www.ncbi.nlm.nih.gov/geo/query/acc.cgi?acc=GSE37182; GSE103512: https://www.ncbi.nlm.nih.gov/geo/query/acc.cgi?acc=GSE103512.

## Abbreviations

CRC: Colorectal cancer
MaRGs: Macrophage-related genes
GEO: Gene Expression Omnibus
WGCNA: Weighted gene co-expression network analysis
CrRGs: CRC related genes
DEGs: Differentially expressed genes
GO: Gene Ontology
KEGG: Kyoto Encyclopedia of Genes and Genes
LASSO: Least absolute shringkage and selection operator
OS: Overall survival
