# Supplementary figures and images for: Identification of macrophage related gene in colorectal cancer patients and their functional roles
Source: BMC Med Genomics. 2021 Jun 13;14:159. doi: 10.1186/s12920-021-01010-0 (PMC8201885; doi:10.1186/s12920-021-01010-0)

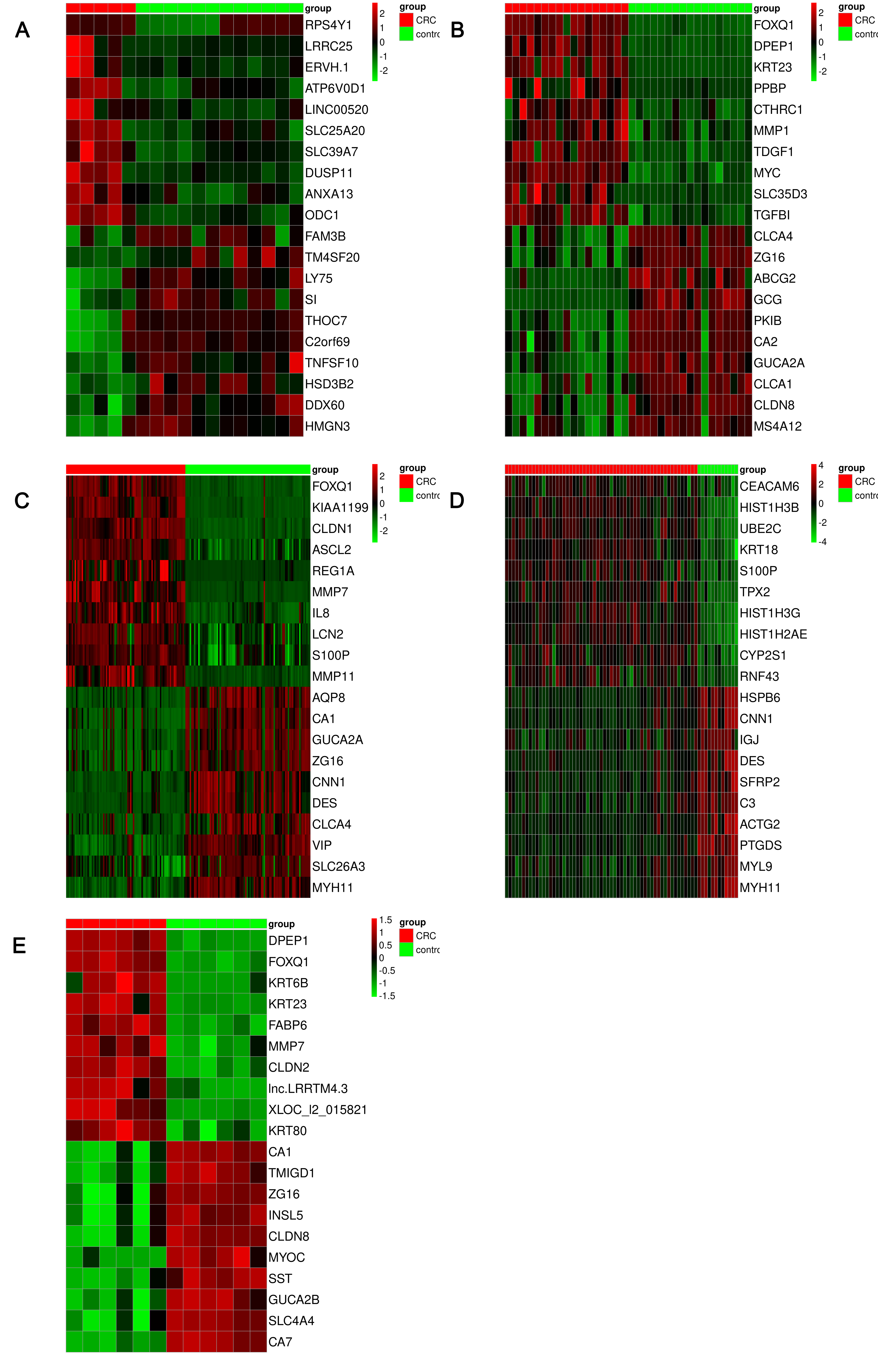

Supplement: Supplementary file 1 — Additional file 1: Fig. S1. Heatmap of the top ten DEGs of up-regulated cluster and down-regulated cluster in five datasets. (A) Heatmap of DEGs identified in GSE23194. (B) Heatmap of DEGs identified in GSE32323. (C) Heatmap of DEGs identified in GSE37182. (D) Heatmap of DEGs identified in GSE103512. (E) Heatmap of DEGs identified in GSE156355 [file 12920_2021_1010_MOESM1_ESM.png]

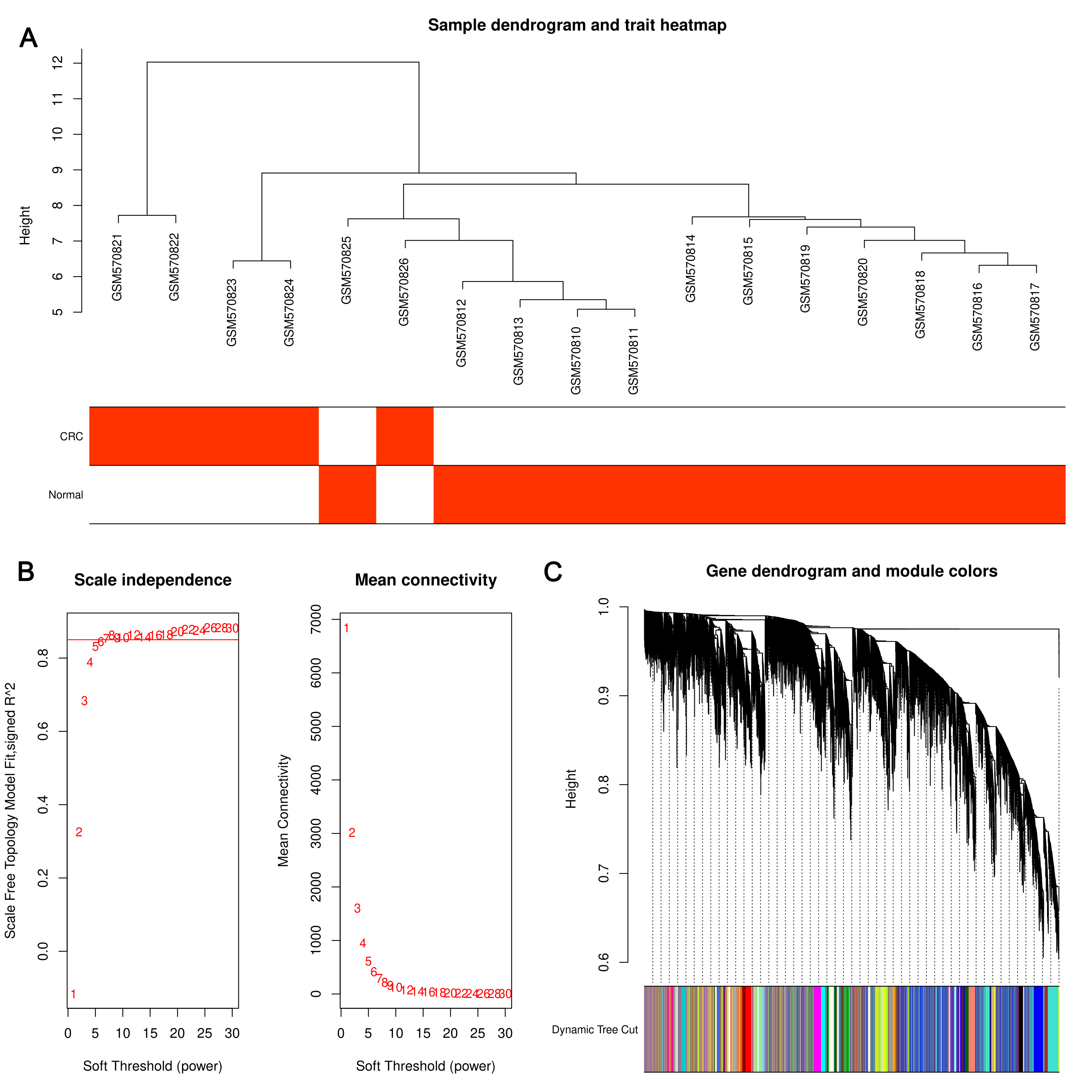

Supplement: Supplementary file 2 — Additional file 2: Fig. S2. WGCNA analysis of GSE23194. (A) Sample dendrogram and clinical trait heatmap. (B) The identification of βvalue for the optimal scale-free topology network. (C) Module identification. The dendrogram indicates the gene clustering according to TOM dissimilarity [file 12920_2021_1010_MOESM2_ESM.png]

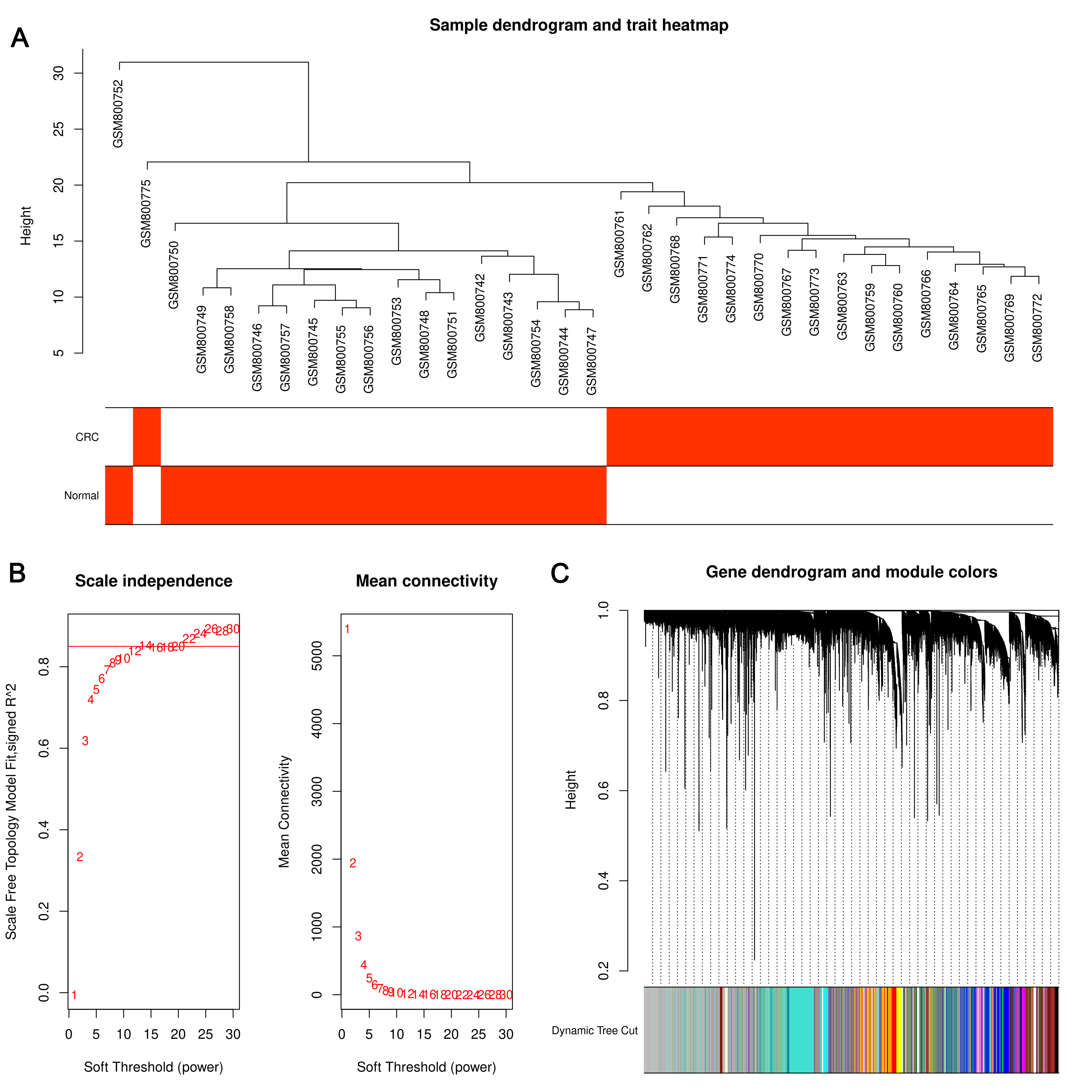

Supplement: Supplementary file 3 — Additional file 3: Fig. S3. WGCNA analysis of GSE32323. (A) Sample dendrogram and clinical trait heatmap. (B) The identification of βvalue for the optimal scale-free topology network. (C) Module identification. The dendrogram indicates the gene clustering according to TOM dissimilarity [file 12920_2021_1010_MOESM3_ESM.png]

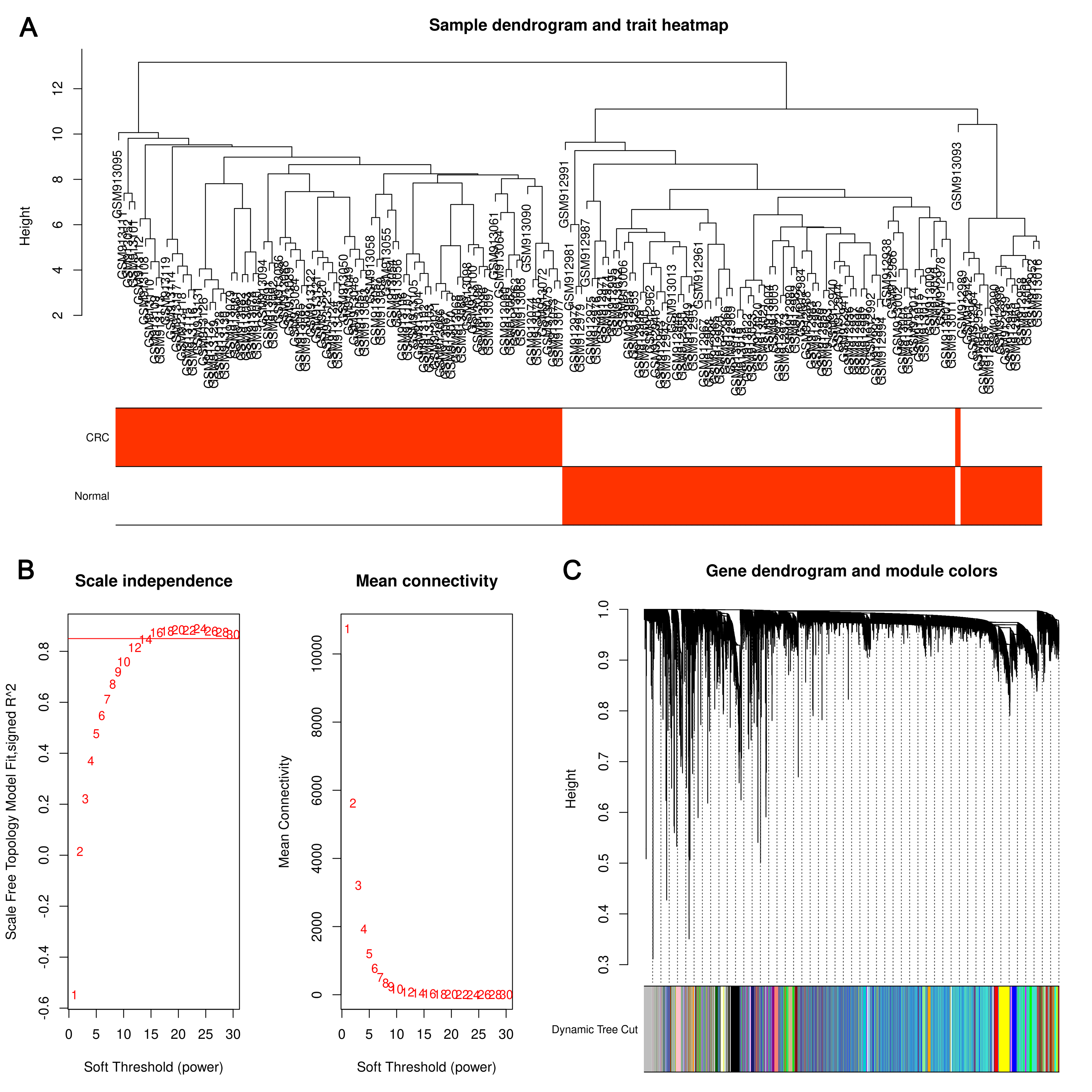

Supplement: Supplementary file 4 — Additional file 4: Fig. S4. WGCNA analysis of GSE37182. (A) Sample dendrogram and clinical trait heatmap. (B) The identification of βvalue for the optimal scale-free topology network. (C) Module identification. The dendrogram indicates the gene clustering according to TOM dissimilarity [file 12920_2021_1010_MOESM4_ESM.png]

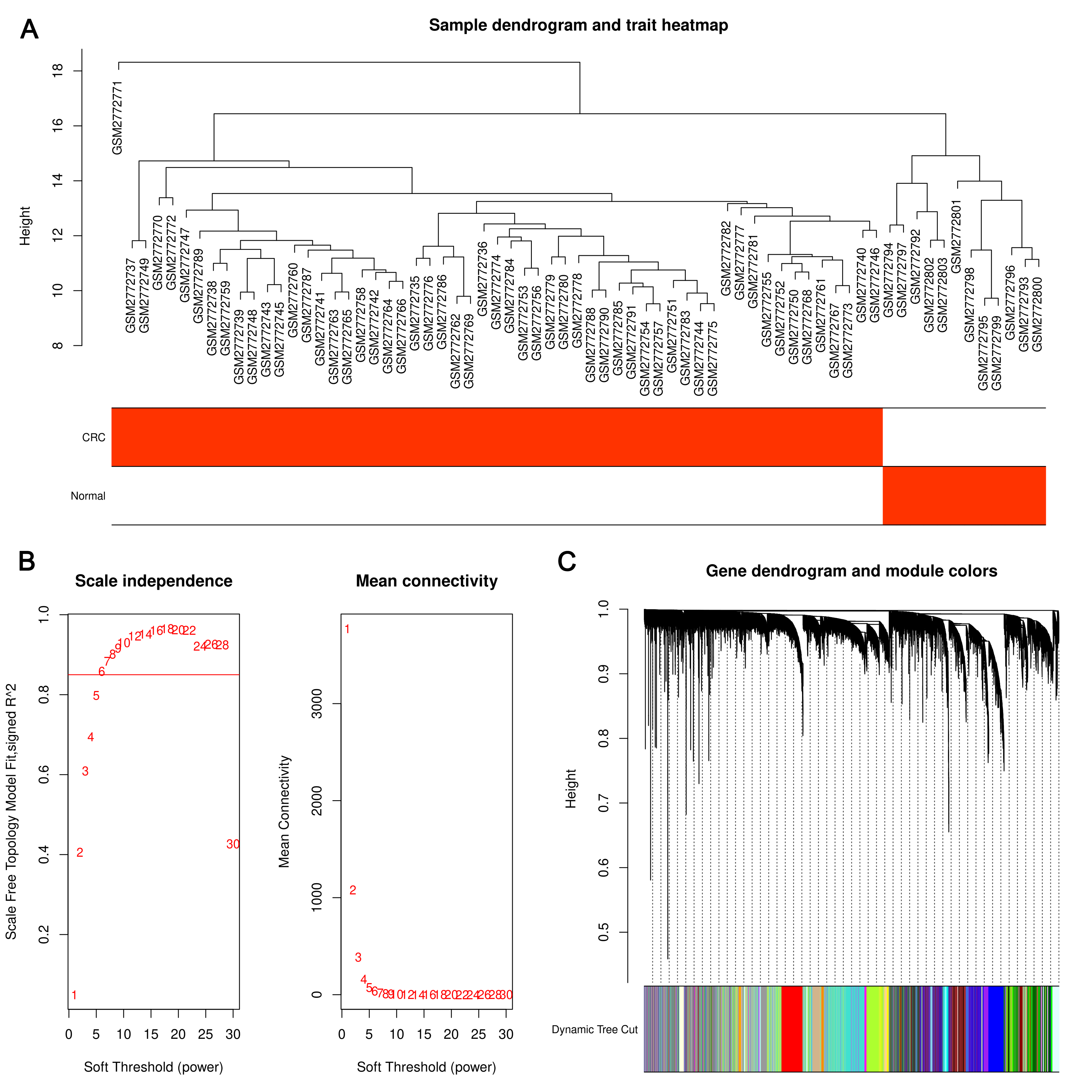

Supplement: Supplementary file 5 — Additional file 5: Fig. S5. WGCNA analysis of GSE103512. (A) Sample dendrogram and clinical trait heatmap. (B) The identification of βvalue for the optimal scale-free topology network. (C) Module identification. The dendrogram indicates the gene clustering according to TOM dissimilarity [file 12920_2021_1010_MOESM5_ESM.png]
